# Drug Metabolism for the Identification of Clinical Biomarkers in Breast Cancer

**DOI:** 10.3390/ijms23063181

**Published:** 2022-03-16

**Authors:** Bárbara Costa, Nuno Vale

**Affiliations:** 1 OncoPharma Research Group, Center for Health Technology and Services Research (CINTESIS), Rua Dr. Plácido da Costa, 4200-450 Porto, Portugal; b.c.211297@gmail.com; 2 Department of Community Medicine, Health Information and Decision (MEDCIDS), Faculty of Medicine, University of Porto, Al. Prof. Hernâni Monteiro, 4200-319 Porto, Portugal; 3 Associate Laboratory RISE-Health Research Network, Faculty of Medicine, University of Porto, Al. Prof. Hernâni Monteiro, 4200-319 Porto, Portugal

**Keywords:** immunotherapy, microbiome, metabolism, pharmacogenomics, pharmacomicrobiomics, biomarkers, precision medicine

## Abstract

Breast cancer is classified into four major molecular subtypes, and is considered a heterogenous disease. The risk profiles and treatment of breast cancer differ according to these subtypes. Early detection dramatically improves the prospects of successful treatment, resulting in a reduction in overall mortality rates. However, almost 30% of women primarily diagnosed with the early-stage disease will eventually develop metastasis or resistance to chemotherapies. Immunotherapies are among the most promising cancer treatment options; however, long-term clinical benefit has only been observed in a small subset of responding patients. The current strategies for diagnosis and treatment rely heavily on histopathological examination and molecular diagnosis, disregarding the tumor microenvironment and microbiome involving cancer cells. In this review, we aim to praise the use of pharmacogenomics and pharmacomicrobiomics as a strategy to identify potential biomarkers for guiding and monitoring therapy in real-time. The finding of these biomarkers can be performed by studying the metabolism of drugs, more specifically, immunometabolism, and its relationship with the microbiome, without neglecting the information provided by genetics. A larger understanding of cancer biology has the potential to improve patient care, enable clinical decisions, and deliver personalized medicine.

## 1. Breast Cancer Therapeutic Options

According to GLOBOCAN 2020 statistics, female breast cancer has exceeded lung cancer as the most diagnosed cancer, having an estimated existence of 2.3 million new cases worldwide [1]. Human breast carcinomas are a heterogeneous disease containing several distinct histological subtypes and four main molecular subtypes; therefore, risk profiles and treatments differ according to these subtypes [2,3]. Breast cancer molecular subtypes are defined by the expression of hormone receptors such as estrogen receptors (ER+) or progesterone receptors (PR+), human epidermal receptor 2 (HER2+), and triple-negative breast cancer (TNBC; which is ER−, PR−, HER2−).

Breast cancer expressing hormone receptors are the most prevalent type; therefore, endocrine therapy is the most employed primary treatment option for breast cancer that is ER/PR-positive [4]. Treatment blocks ER activity either with antiestrogens, such as Tamoxifen or Fulvustrant, or by weakening ER-mediated signaling by lowering estrogen synthesis with aromatase inhibitors [5]. Aromatase inhibitors, such as Letrozole, are preferable to Tamoxifen as a first-line treatment in menopausal women [6].

HER2-positive breast cancers are described by aggressive disease progression and poor prognosis. Targeted therapies for HER2-positive tumors comprise trastuzumab or pertuzumab, anti-HER2 monoclonal antibodies, lapatinib, a small-molecule tyrosine kinase inhibitor or trastuzumab emtansine (T-DM1), an antibody-drug conjugate [7].

Triple-negative breast cancer (TNBC) is the most challenging form of this cancer. Its clinical feature consists of high invasiveness, high metastatic potential, relapse proclivity, and poor prognosis. Antiestrogens and anti-HER2 therapeutics are sometimes unsuccessful in treating TNBC; however, the use of anthracycline and taxane-based chemotherapy is still the norm for early-stage TNBC. The inclusion of carboplatin to the ACT (anthracycline, cyclophosphamide, and taxane) regimen is linked to a better complete pathologic response (pCR). TNBC tumors lack known targets for effective therapies, and treatment options are limited to chemotherapy. Recently, combinations of immune checkpoint inhibitors (ICIs) have dramatically boosted pCR in TNBC. Other targets include inhibitors of the Phosphatidylinositol-3-kinase/Protein Kinase B/mammalian target of rapamycin (PI3K-AKT-mTOR) pathway and poly-ADP-ribosyl polymerase inhibitors (PARPi) [3,8].

According to the World Health Organization, breast cancer treatment can be highly effective, with a 90% or higher chance of survival, mainly when the disease is detected early. Breast cancer survival for at least 5 years after diagnosis varies from more than 90% in high-income countries to 66% in India and 40% in South Africa. Surgery or mastectomy, with or without radiation, is the standard treatment option for localized breast cancer. At the same time, systemic adjuvant therapies are used to monitor tumor growth and improve survival. The rapid expansion of antineoplastic drugs has its advantages, since it means the accessibility of more therapeutic options. There are also disadvantages, representing a challenge to oncologists who must select the best treatment for every patient. Usually, oncologists are dependent on the best evidence to decide on the most suitable anticancer treatment. However, two problems emerge: (1) only a fraction of patients respond to any chemotherapy regimen using this approach, and (2) the portion of responding patients decreases with successive lines of chemotherapy due to the emergence of resistance [9]. Drug doses (or a combination of drugs) are selected based on the maximum tolerated amount. Dose intensity is modified as toxicities appear; however, such adverse effects tend to be cumulative and can be life-threatening or debilitating, impacting quality of life. The oncologist evaluates treatment efficacy only 2–3 months after the patient begins chemotherapy; in the meantime, substantial toxicities may have developed, and high treatment-related costs may have accrued while there is no knowledge of whether the patient is benefiting or not. The treatment effect for solid tumors, such as breast cancer, is typically seen as a reduction in tumor size. Repeated radiographic examinations are costly and time-consuming, and tumor development also emerges in a delayed form, frequently after clinical worsening. Additional breakthroughs in breast cancer treatment have contributed to the development of specific biomarkers for patient selection and treatment response prediction, resulting in minimal drug response failure. However, while predictive indicators may help with therapy selection, most treatments lack predictive biomarkers. They only recognize those who do not respond to certain drugs; thus, there is no promise of benefit due to the need for extensive validation studies before clinical use [9].

In contrast to conventional treatments that lack tumor selectivity and cause more side effects, immunotherapy and other treatment strategies targeting tumor cells are promising options. Immunotherapies work by enhancing a person’s immune system’s ability to recognize and destroy cancer cells. The ability of the immune system to prevent itself from attacking normal cells in the body is an important part of its function. It accomplishes this by utilizing proteins (or “checkpoints”) on immune cells that must be activated (or deactivated) in order to initiate an immune response. These checkpoints are sometimes used by breast cancer cells to avoid being attacked by the immune system. Because the immune system has already recognized this cancer, the pre-existing response can be improved with immunotherapies, enhancing breast cancer immunogenicity. Breast cancer is less immunogenic than other tumor forms; nonetheless, anti-PD1/PD-L1 drugs have been studied in breast cancer in recent years, specifically in the triple-negative subtype, with promising results when used alone or in combination with other treatments [10]. Immune cells play an essential role in breast cancer recognition and early eradication and tumor progression. Immunoediting is a three-step process that describes the interactions between the host’s immune system and the tumor’s cells. It includes elimination, equilibrium, and escape. Recently, a fourth phase called exhaustion has been proposed. Immunotherapy targets specific proteins to stimulate the immune system to detect and remove cancerous cells. The impact of immune-checkpoint inhibitors (ICPIs), anti-tumor vaccinations, the transmission of elective T-cell treatment, and monoclonal antibodies (mAb)-based immunotherapy are some of the tactics being used. Immunotherapy is a relatively recent kind of breast cancer treatment, even though breast cancer is not one of the most immunogenic malignancies, such as melanoma and lung cancer. Recent clinical trials have documented that PD-1/PD-L1 inhibitors alone have little efficacy, but when used in combination with other treatments clinical efficacy increases. Therefore, some mAb have been used as therapeutic agents, with Trastuzumab as an example of passive immunotherapy [11]. In recent years, additional modalities, such as antibody-drug conjugates (ADC) and ICIs, have been studied as a prophylactic and treatment approach for breast cancer [10].

Some patients do not respond to initial immunotherapy, despite increased rates of benefit and survival in tumor patients. Treatment resistance (which corresponds to primary and acquired resistance, respectively) causes some responders to relapse or progress after a treatment period [12]. Adaptive resistance is a new type of resistance that has been recently proposed. The tumor can adapt to the immune attack by changing itself, which can happen as a primary or acquired resistance. Immune resistance in tumors is caused by the intertwine of gene expression TME, metabolism, aberrant neovascularization, inflammation, etc. Identifying and utilizing biomarkers to guide predictive decision insights for expanding immunotherapy applications is essential. Companion biomarkers such as PD-L1 immunohistochemistry (IHC) and tumor mutational burden (TMB), in addition to ICPI, have received regulatory approval for a variety of applications. Pembrolizumab was the first FDA-approved drug based on tumor microsatellite instability/deficient mismatch repair (MSI-H/dMMR) biomarker status rather than tumor histology. Several other ICPI-related biomarkers are being investigated, including tumor-infiltrating lymphocyte (TIL) measurement, gene expression profiling in an inflammatory microenvironment, and prediction of neoantigen. TMB and PD-L1 expression can predict a subset of responses to checkpoint blocking, but not all reactions. Even though no single biomarker can faithfully reproduce the complexity of the tumor-immune microenvironment, a selected combination of biomarkers should emerge as a method to improve predictive power [13,14].

## 2. Immunometabolism

### 2.1. Limitations of Breast Cancer Subtyping

For patient stratification in breast cancer, several clinical factors such as menopausal status, age, lymph node invasion, and tumor size are combined with gene expression-based signatures and subtyping systems. These are widely recognized as valuable methods in determining the most effective treatment option for a breast cancer patient. However, traditional subtyping has limitations because it ignores interactions with the tumor microenvironment (TME). The highly inflammatory microenvironment with infiltrating immune cells, cytokines, and growth factors differentiates breast cancer [15]. The TME influences energy consumption and metabolic reprogramming in immune cells, promoting angiogenesis, which restores the oxygen and nutrient supply while removing metabolic waste to overcome a hypoxic and acidic microenvironment. Since tumors are infiltrated by a variety of adaptive and innate immune cells, the traditional therapy efficacy can be altered by the TME’s pre-existing inflammatory and stromal cells and modify it [16].

Immune cells in the TME can either promote an anti-tumor or a pro-tumor microenvironment. An anti-tumor microenvironment refers to the innate and adaptive immune responses that result in tumor control. T cell priming against tumor antigens, trafficking of these anti-tumor T cells to the tumor tissue, T cell infiltration and local activation to kill tumor cells, and appropriate resolution of the immune response, including clearing of the lysed tumor cells and re-establishment of normal tissue architecture and homeostasis, are all required for successful anti-tumor immunity. The most powerful mediators of the adaptive anti-tumor immune response are T cells. The final effector mechanism leading to tumor elimination is produced by the cytotoxic CD8+ T cell population, which is supported by CD4+ T helper (Th1) cells through the production of IL2 and IFN and is associated with a positive prognosis. Pro-tumor immunity, on the other hand, suppresses anti-tumor adaptive and innate responses and promotes tumor development. Chronic inflammation upregulates several immune pro-tumor effector mechanisms, leading to the theory that inflammation promotes carcinogenesis and tumor growth by disrupting the balance between pro- and anti-tumor immunity, preventing the immune system from rejecting malignant cells and creating an environment suitable for disease progression. Tumor progression is assisted by CD4+ T regulatory cells, type 2 CD4+ T cells, type 2 natural killer T cells, myeloid-derived suppressor cells, M2 or tumor-associated macrophages, B cells, and possibly mast cells, whereas tumor destruction is supported by CD8+ T lymphocytes, type 1 CD4+ T lymphocytes, natural killer, type 1 natural killer T cells, M1 macrophages, and immune killer dendritic cells [16].

Immune infiltration brings a specific signature to each subtype of breast cancer. Regulatory T cells (T regs) do not provide much additional information since they confer an immunosuppressive environment in both ER-positive and ER-negative tumors and are generally associated with poor prognosis. Natural killer cells (NK) and neutrophils are abundant in ER-positive breast tumors, while TCD8+ cytotoxic T cells, TCD4+ naïve, and memory T cells are observed in smaller proportions. Moreover, eosinophils and monocytes have been linked to a good chemotherapy response, and B lymphocytes have been linked to a good prognosis in this phenotype as well. Because of their immunosuppressive, pro-tumorigenic and inflammatory purposes, tumor-associated macrophages (TAMs) 1 and 2 and T reg lymphocytes give a poor prognosis. T regs, TAM2, and activated mast cells are the most common immune infiltration cells in ER-negative breast tumors, and they are also linked to a poor prognosis. TCD4+, TCD8+, dendritic cells (DCs), and B lymphocytes, on the other hand, are associated with a better prognosis but are found in lower numbers and can be linked to a favorable response to neoadjuvant chemotherapy. There are few reports of infiltrating immune masses in HER2-positive breast cancer; however, they are primarily represented by DCs, mast cells, neutrophils, T lymphocytes, and T regs. This is all associated with a poor prognosis, metastasis, and disease relapse [17], see Table 1. 

Moreover, depending on the concentration of TILs it is possible to correlate varying sensitivity to therapeutics. Therefore, is possible to classify tumor and therapies according to stromal TILs concentration. HER2+ and TNBC are more likely than luminal breast cancer to have stomal TILs at diagnosis [18]. The immune infiltration of breast tumors change the clinical outcome since it modulates treatment response. Again, TNBCs and HER-2+ breast cancers are also more likely to express the programmed death ligand-1 (PD-L1) than luminal breast cancers in the TME. TIL levels at diagnosis predict adjuvant and neoadjuvant treatment benefit, as well as prolonged progression-free survival (PFS) and overall survival (OS). TIL-positive solid tumors with PD-L1 expression are more likely to react to PD-1/PD-L1 inhibition [19,20]. These immune cell patterns establish a relationship between the heterogeneity of immune infiltrating cells, the tumor phenotype, and the treatment response in breast cancer. Thus, all of this should be considered for breast cancer subtyping and patient stratification for personalized medicine.

Tumor cells use growth factors, cytokines, and chemokines to attract stromal cells (e.g., fibroblasts), immune cells, and vascular cells. By releasing growth-promoting signals and intermediate metabolites, as well as remodeling tissue structure, these cells help to create the microenvironment. Proliferation and metastatic ability are stimulated or inhibited by signaling between cancer cells and the TME. The tumor not only manages to evade the host immune system, but it also exploits infiltrating cells by altering their functions to create a favorable microenvironment for tumor progression [21].

Furthermore, the relationship between cellular and non-cellular components must be acknowledged since they are vital regulators of primary tumor progression, organ-specific metastasis, and therapeutic response. The extracellular matrix (ECM) is the main non-cellular component of the TME, and it undergoes significant changes during tumorigenesis to aid malignant cell growth and survival [22]. Large ECM deposits in solid tumors can account for up to 60% of the tumor’s bulk. Large collagen deposits in conjunction with a high-level percentage of fibroblast infiltration are strongly associated with a poor patient prognosis. Proteases cause the release of cytokines and growth factors that are stored in the ECM [23]. Proangiogenic factors, including VEGF, FGF, PDGFB, and TGFB, can be found in the ECM [22].

Another non-cellular component is the Exosomes (microvesicles that range from 30 nm to 200 nm released by normal and tumor cells) that play a critical role in facilitating crosstalk between cancer cells and stromal cells within the TME [24]. Their contents reflect the original cell, including protein, DNA, RNA, and lipids, and they have been proven in the TME to induce inflammation, tumor development, angiogenesis, and metastasis. Hypoxia appears to exacerbate cancer cell exosome production and stimulate the transformation of stromal cells into cancer-associated fibroblasts (CAFs). Exosomes derived from tumors that contain tumor-specific antigens and nucleic acids can be evaluated non-invasively as a diagnostic and predictive biomarker. Furthermore, exosomes can be applied for the identification of patients at risk of developing metastatic disease. The process of exosome production could lead to new cancer therapy targets [24].

To alleviate oxygen shortage and accumulate metabolic waste, growing tumors require the development of new blood vessels; thus, inhibiting angiogenesis was an appealing technique. The VEGF/VEGFR signaling axis has been the focus of antiangiogenic (AA) therapy, which has comprised: Bevacizumab, a neutralizing antibody to VEGF-A; Aflibercept, a decoy receptor for VEGF-A or B; Sorafenib, a tyrosine kinase inhibitor; and Aflibercept and Ramucirumab antibodies that block VEGF, binding its receptor. Most patients do not respond to AA therapy as a single agent or develop resistance. The successful integration of AA therapy into the clinic will almost certainly necessitate other drugs [25]. Even though therapeutically targeting the TME is an appealing strategy for cancer treatment, existing FDA-approved treatments are ineffective.

Understanding the TME, its activities, and related chemicals in depth will provide vital insights into the biological behavior of distinct tumor types, because of its probable function in carcinogenesis. It can be utilized to control tumor cells, affecting tumor start, development, and advancement in a variety of ways. TME cells and chemicals can boost tumor cell stemness, stimulate angiogenesis, mediate migration, cause treatment resistance, and inhibit the immune system [22]. Taking all of this into account, the immunological TME evaluation has crucial predictive value and can enhance histopathological and molecular indicators in evaluating patient responses to treatment.

### 2.2. Metabolism Regulates Immune Cell Activation

The metabolic requirements of immune cells in the TME influence the success of anticancer immunotherapies. Either the use of radiotherapy or chemotherapy induces tumor cell death, increasing the availability of nutrients in the TME which appears to be crucial for immune cell activation and function. The Warburg effect is a hallmark phenomenon found in cancer cells that leads to increased glycolysis and decreased mitochondrial oxidation, which supports the high proliferative capacity of cancer cells. Reversing Warburg metabolism can decrease breast cancer cell proliferation or prevent cell death. More metabolic pathways shown to be upregulated in breast cancer are glutamine metabolism, the glutamine-serine pathway, lipid and fatty acid, cholesterol metabolism, and protein translation [26]. The activation of AA, mTOR, estrogen-related receptors, PI3 kinase, AMPK, estrogen receptors, peroxisome proliferator-activated receptor cofactor-1α and β (PGC1α and PGC1β), or nuclear respiratory factor 1 (NRF1) represent the complex reorganization of the cellular energy network [27].

All breast cancer subtypes have metabolic changes, and there is solid evidence that variations in metabolism can be used to distinguish between the intrinsic subtypes. Glycolytic metabolism in immune cells usually take the lead to activation of an effector phenotype, for example DC, NK, B cells [28]. In contrast, the oxidative metabolism of substrates such as fatty acids and amino acids, including glutamine, leads to a regulatory or memory phenotype [29]. T cells, for example, are stimulated via the antigen receptor, and CD28 costimulation can increase the expression of the glucose transporter GLUT1, glucose absorption and subsequent glycolysis, and mitochondrial capacity. This metabolic shift can be suppressed by CD28 inhibitory receptors such as cytotoxic T-lymphocyte-associated protein 4 (CTLA4) and PD-1 [30]. Memory T cells rely on mitochondrial metabolism and lipid oxidation rather than aerobic glycolysis, as effector T cells do. Due to direct linkages between the endoplasmic reticulum and mitochondria, which serve as metabolic hubs, they can swiftly revert to glycolysis upon restimulation. Tregs, unlike effector T cell subsets, do not require GLUT1 or large amounts of glutamine uptake through the amino acid transporter ASCT2, instead relying on lipid, pyruvate, and lactate oxidation in the mitochondria. Tregs can be quite glycolytic, although FoxP3, the main Treg transcription factor, has been found to suppress glycolysis. Treg suppressive capacity can be harmed by high glucose metabolism rates [31].

The mTOR pathway’s mechanistic target is important in immunometabolism and cell fate. mTOR is a component of two protein complexes that are activated by PI3K and Akt receptor activation. The PI3K/Akt/mTORC1 pathway is an important mechanism for sensing and integrating food availability and signaling in order to promote T cell metabolism and function. mTORC1 is activated at lysosomes by the combined effects of T cell activation and signaling via PI3K and Akt, which leads to the activation of the Rheb GTPase and coordinated amino acid sensing via the sestrin/GATOR complex [32].

The landscape of metabolic changes is more complicated due to differences in the metabolism of breast cancer cell types. Furthermore, cancer stroma cells use Warburg metabolism, whereas cancer stem cells’ metabolism is driven by mitochondrial oxidation [33]. In breast cancer, there are two types of cancer stem cells: CD44+/CD24 mesenchymal-like cancer stem cells and aldehyde dehydrogenase 1 family, member A1 (ALDH1) positive epithelial-like cancer stem cells [34]. Inhibiting mitochondrial oxidation can increase the proportion of cancer stroma to stem cells, allowing conventional chemotherapy to be more effective against stromal cells. TNBCs contain a higher percentage of stem cells than ER+ cancers [35]. This observation is supported by the fact that the TCA cycle is more active in TNBC than in ER+ cases [36]. There is growing evidence that circulating cancer cells’ metabolism shifts to oxidative phosphorylation [37].

Discovering new approaches to understanding the variety of metabolic programs in tissue-specific areas will lead to novel discoveries and immune regulation options. While existing medicines, such as methotrexate, can target metabolic pathways, a promising comprehension of cell and tissue biology will surely discover new targets with greater specificity and lower toxicity.

### 2.3. Drug-Induced Alterations

Resistance to several anticancer drugs is associated with the increased activity of redox balance pathways, indicating that intervening with redox metabolism can improve drug response and help overcome multidrug resistance. Changes introduced by drugs in the circulating and intratumorally metabolomes result from pharmacological effects. Metabolomics’ pharmacokinetics and pharmacodynamics can be used to evaluate the efficacy and side effects of breast cancer treatment. Regardless of the type of chemotherapeutic agent used, increased glycolysis is a common feature of drug-resistant breast cancer cells. However, this increased activity is regulated in different ways in different resistant breast tumors. Therefore, metabolic profiles are altered according to the drug being used.

The Warburg effect has been linked to drug resistance in several studies, meaning that a high glycolytic rate helps cancer cells survive anticancer drugs such as bortezomib, cisplatin, and lapatinib [38]. Because some medications are unstable in acidic environments, higher glycolytic rates have been postulated to limit therapeutic efficacy by increasing lactate output and acidification of the extracellular space. Glycolytic regulators such as PDK1 and LDHA are typically overexpressed in drug-resistant cells, making them potential targets for drug-resistant malignancies. Lapatinib-resistant SKBR3 breast cancer cells expressed more genes associated with glucose deprivation than sensitive cells, which was connected to a worse patient outcome. Glucose transporters and glycolytic enzymes were among the genes identified, as were alternative energy production pathways such as oxidation [39]. In trastuzumab-resistant ErbB2-positive breast cancer cells, higher glycolytic activity is mediated by heat shock factor 1 and LDHA. Furthermore, inhibiting glycolysis with 2-DG and the LDH inhibitor oxamate resensitizes trastuzumab-resistant cells [40]. Finally, when LDHA was genetically downregulated or paclitaxel was combined with oxamate, synergistic effects on inducing apoptosis were evidenced in paclitaxel-resistant breast cancer cells [41].

The nuclear receptor estrogen-related receptor (ERR) alpha allows breast cancer cells to use lactate as a substrate for mitochondrial respiration when glucose is not present. They gain the ability to circumvent glycolysis which makes these cells less vulnerable to PI3K/mTOR inhibitors, and ERR antagonists can restore drug efficacy [42,43]. By reactivating mTOR signaling, breast cancer cells resistant to lapatinib restore ERR levels, resulting in increased glutamine metabolism, antioxidant capacity, and mitochondrial energy production. Furthermore, targeting ERR counteracts the metabolic alterations associated with lapatinib resistance and overcame resistance to this drug in an HER2-induced mammary tumor mouse model [42]. Therefore, targeting ERRα could reduce tumor resistance to therapy administered by increasing the sensitivity of drug-resistant breast cancer cells in the context of metabolism.

Another metabolic trait observed in drug-resistant breast cancer cells is higher levels of OXPHOS, as well as increased levels of oxidative stress. For example, tamoxifen-resistant MCF-7 breast cancer cells have a higher rate of mitochondrial metabolism, and ATP production and metformin selectively kill breast cancer stem cells resistant to standard chemotherapy, highlighting the importance of OXPHOS activity in drug response. Because tamoxifen-resistant breast cancer cells are more likely to be subjected to oxidative stress, this higher mitochondrial activity could possibly explain why these cells have lower GSH levels. Tamoxifen-resistant cells exhibit greater amounts of the oxidative stress defense enzymes NADPH dehydrogenase 1 (NQO1) and GCLC.

Furthermore, transduction of these genes into MCF-7 cells causes a tamoxifen-resistant phenotype, with NQO1 mRNA levels linked to disease progression in endocrine therapy patients. As a result, tamoxifen sensitivity was restored in tamoxifen-resistant breast cancer cells after NQO1 inhibition with dicoumarol. The inhibition of GSH biosynthesis with BSO synergized with cisplatin induced regression in PI3K/Akt driven breast cancer [44]. Increased GSH synthesis was also observed in PI3K/Akt-driven breast cancer and was required to resist oxidative stress. These findings suggest that increased antioxidant defenses in breast cancer drive resistance to various chemotherapy types.

Furthermore, fatty acid synthesis (FASN) is associated with a bad prognosis in various forms of cancer and impairs therapeutic efficacy. FASN overexpression induces resistance to the anticancer medicines adriamycin and mitoxantrone in breast cancer cells. Orlistat, a FASN inhibitor, enhances medication sensitivity across the board, implying that FASN could be a potential target in treatment-resistant malignancies [45] (see Table 2). The branched-chain amino acids, such as serine, proline, aspartate, and arginine are also linked to carcinogenesis. The significance of amino acid metabolism in drug resistance is mainly understood, but research indicates that amino acid availability may play a role in therapeutic responsiveness and drug resistance development.

Hence, different types and stages of cancer may rely on different metabolic pathways. Different types of drugs and their combination will have different results in efficacy and toxicity in different patients. Identifying tumors and patients resistant to treatment early in the regimen requires a quick and accurate response to therapies [46]. The development of biomarkers that predict response and resistance to therapy, plus the identification of environmental modifiers of immunity (microbiome, metabolic and hormonal parameters, and concurrent drug therapy), are all areas of research that are gaining traction.

## 3. Finding Biomarkers

Biomarkers help reveal connections between environmental exposures, human biology, and disease. Biomarkers can help scientists better understand fundamental biological processes, advance exposure science, and translate research findings into medical and public health applications. The 1970s was the first-time biomarkers in breast cancer were used to treat the disease, and tumors expressing ER+ were treated with tamoxifen. Alterations in the genes ERBB2, ER, PR, BRCA1, BRCA2, and PIK3CA are currently clinically actionable genomic abnormalities with FDA-approved drugs. However, inter-individual variation in treatment response is a complicating factor that has a severe influence on patient health and imposes significant clinical and financial costs. Response rates to typical medications used to treat a variety of disorders have been estimated to be in the 50–75 percent range, meaning that up to half of patients receive no benefit. Patients who initially respond to anti-HER2 drugs often develop secondary resistance within one year after treatment initiation, while those who show primary resistance to HER2-targeted therapy can still benefit from anti-HER2 regimens [47].

Biomarkers are especially useful in identifying individuals at an increased risk of developing breast cancer within high-risk families. However, the existence of biomarkers with the capacity of determining prognosis, determining the most suitable drug treatment, monitoring capacity, and post-operative follow-up are essential to increase the success of treatments and reduce relapses. Tumor size and tumor grade are two significant prognostic indicators that are commonly employed. However, there are limits in tumor grading, such as lack of reproducibility and tumor heterogeneity. Since we are in the era of individualized medicine, these prognostic biomarkers (tumor size, tumor grade, and lymph node metastases) are insufficient for the proper therapy of patients with early-stage breast cancer [48]. Hundreds of potential biomarkers for predicting outcomes in women with newly diagnosed breast cancer have been proposed. However, most of these studies had minimal evidence [49] due to small patient populations, lack of independent prognostic value, insufficient clinical validation, poor design, and inability to demonstrate clinical relevance.

Biomarkers are categorized according to their clinical value (Table 3). Cancer biomarkers range from macromolecules such as DNA, RNA, proteins, to whole cells. Breast cancer biomarkers have been identified for cancer risk, diagnosis, proliferation, metastasis, drug resistance, and prognosis due to the growing demand for personalized or precise treatments. Identifying novel biomarkers is essential in modern age medicine to aid in the development of new drugs, assist therapies, monitor therapy efficacy, and ensure survival with a good quality of life.

At the time of breast cancer diagnosis, an accurate determination of prognosis is essential for optimal patient management, particularly to escape the overtreatment of unaggressive disease and the undertreatment of aggressive forms. Most breast cancer patients are diagnosed early enough to benefit from surgery, chemotherapy, radiotherapy, or a combination of these treatments. Nevertheless, it is considered that 30% of women who are initially diagnosed with early-stage disease will eventually develop metastases [49,50], which eventually increases morbidity and mortality.

Response biomarkers based on blood or urine testing are less expensive and invasive than radiographic methods. Furthermore, assuming a response biomarker is an early indicator of benefit or resistance, it will make it easier to decide whether the patient should continue with any drug or drug combination in relatively early stage, before toxicities appear and clinical deterioration occurs. A response biomarker that responds fast after treatment begins does not require a high pre-treatment baseline level; it is more generalizable to diverse tumor types and medications would be the ideal choice [49]. A good biomarker should be specific, sensitive, and low-cost, with high throughput assay ability.

Biomarker research is also evolving to include a combinatorial approach to identifying biomarkers from multi-omic data. The ability to develop panels that evaluate treatment response based on many biomarkers at once can be achieved by combining biomarkers from various omic data groups. In this review, we aim to explore pharmacogenomics and pharmacomicrobiomics to integrate them in patient care and facilitate tumor categorization.

### 3.1. Pharmacogenomics, Defining a Metabolic Background of Tumors: Genome Take on Drug Metabolism

Hypoxia, inflammation, and alterations in metabolism can be drivers of carcinogenesis, implying that cancer is a metabolic illness that can be caused by genetic or non-genetic signaling and metabolic abnormalities [51]. Because it was previously simple to identify and characterize genomic and transcriptomic changes in cancer, tumors were mostly identified using genomic and transcriptomic signatures, with metabolic profiles being rarely employed. Thanks to recent technological breakthroughs in metabolomics, it has become simpler to understand the specific contribution of disordered metabolism alongside genomic and transcriptomic abnormalities. It is critical to consider their contribution because metabolic pathways are highly adaptable and can be remodeled in response to tissue context, tumor architecture, and the TME. Furthermore, while significant progress has been made in considering the regulation and requirements of metabolic processes in tumor cells and TME cells, identifying the dynamic metabolic interactions between different cells in vivo has yet to be accomplished. As a result, multimodal strategies must be used to collect data from cancer patients before, during, and after treatment. Genetic mutations altered transcriptional indicators, and metabolic shifts can all be used to help identify synthetic lethal pairs or drug combinations for specific targets. To detect and visualize the in vivo activity of metabolic pathways, new imaging and analytical approaches must be developed.

Pharmacogenomics was created to investigate the combined effects of genetic differences on pharmacological action (pharmacodynamics) and disposition (pharmacokinetics) in individuals [52], i.e., how an individual’s genetic makeup influences their medication reaction. PharmGKB (https://www.pharmgkb.org/, last accessed in 10 February 2022) is a pharmacogenomics knowledge resource that provides gene–drug relationships as well as detailed guidelines for using pharmacogenomics in clinical practice [53]. Several international scientific consortia have issued pharmacogenetic guidelines in recent years, but their use in clinical practice is still limited. Worldwide collaborations are underway to overcome present barriers to pharmacogenomic application. On the other hand, existing validated pharmacogenomic markers can only explain a small portion of the detected clinical variability in therapeutic outcomes. New research methodologies, such as researching the immune system’s response to immunotherapy to detect previously undetected uncommon genetic variations (which have been revealed to account for a considerable amount of inter-individual variability in drug metabolism) are required.

Despite the broad use of ER, PR, and HER2 as biomarkers in treatment, these genes are not the most frequently mutated in breast cancer. The most important clinical application of ER expression is as a predictor of endocrine treatment response. Estrogens are thought to promote cancer cell proliferation through their interactions with regulatory components in the genome such as cyclin D and MYC [54]. Because estrogens exert their effects via the ER, it is hypothesized that ER levels may be connected to the therapeutic effects of antiestrogenic treatment. The HER2 marker is another prognostic biomarker that must be evaluated in conjunction with ER in all newly diagnosed individuals. High HER2 expression promotes metastasis, invasion, and the proliferation of cancer cells via the activation of signaling pathways such as PI3K/AKT and MAPK, as well as cell membrane deformation [55]. Several attempts have been made to develop and validate a novel approach for breast cancer patients. Breast cancer tissue has been found to express single or many genes, as well as single or multiple micro-RNAs (mi-RNAs) [56]. Furthermore, circulating tumor DNA (ctDNA) and circulating tumor cells (CTCs) have been discovered in peripheral blood mi-RNA expression. Many researchers have been working on CTCs, miRNAs, and DNA mutation testing (such as ctDNA measurement) to uncover novel prognostic and predictive markers in recent years [57].

Amplification of the MYC oncogenes, mutations in the RAS family, and changes of tumor suppressor genes such as TP53 (mutated in 50% of malignancies) are difficult to target directly, and there are currently no FDA-approved drugs [58]. Moreover, as molecular data grows more complex, so does the corresponding information for clinical diagnosis. Gene expression profiling measures the expression of a thousand genes at once; however, it does not meet the description of a companion diagnostic. The results are used to stratify patients for expected response to standard chemotherapeutic regimens; however, they do not necessarily indicate a specific targeted therapy. Companion diagnostics are most understood and used in conjunction with specific molecular genomic discoveries in oncology patients who are likely to react to targeted therapy. For example, Her-2 amplification/overexpression is the most important biomarker for trastuzumab treatment in breast cancer [59]. However, it is necessary to proceed with caution when used alone since multiple genetic variants might alter the response to a single therapy (e.g., MDM2 amplification and TP53 variants affecting MDM2 antagonists) and a single biomarker might indicate multiple therapies (e.g., estrogen ablation or antagonism based on ER expression). Tumor genome profiling gives a glimpse of a tumor’s genetic complexity, but this information is insufficient to guide therapy for most patients. Therefore, we need multiple biomarkers to guide all cancer treatment processes.

Among the most widely investigated biomarkers, we can find gene expression profiles (e.g., Oncotype DX, MammaPrint), Ki-67, urokinase plasminogen activator (uPA)/PAI-1, and the serum biomarkers: CA 15-3 and CEA [57]. Oncotype DX is a validated and widely used multigene signature test for predicting the risk of recurrence in ER+ lymph node-negative breast cancer patients treated with adjuvant Tamoxifen [60]. MammaPrint is another validated molecular test that employs microarrays to assess the relative expression of 70 genes that are mostly implicated in cancer regulatory pathways [61]. Predicting the likelihood of cancer recurrence is an important therapy marker. These multigene signature tests are not cheap, being excessively expensive in many countries. To set up a simple and inexpensive test to serve as a diagnostic and predictive biomarker test, considerable efforts have been devoted. Ki67 and IHC4 are some of those economical biomarkers, with Ki67 being the popular choice [62]. Considering that using tumor tissue can be a limitation, circulating biomarkers that are robust and clinically verified are advised. TPS, CEA, and CA 15-3 are examples of circulating biomarkers with increased levels [57]. Apart from their potential function in predicting poor outcomes in breast cancer patients, these biomarkers do not currently meet the criteria to be used in clinical practice as prognostic biomarkers. Biomarkers based on gene expression levels are more susceptible to batch effects, vary considerably between cell types, and are affected by gene expression levels in both cancer and non-cancer cells. On the contrary, pathway-level techniques are more resistant to batch effects, allowing for data aggregation from multiple cohorts and the identification of highly important biomarkers [63]. However, owing to a lack of large-scale gene expression data from a variety of cancer cell lines, as well as their susceptibility to a wide collection of anticancer treatments, identifying pathway-based expression indicators has proven problematic in the past. However, recent developments in gene silencing and editing have permitted the creation of large-scale gene essentiality data in hundreds of cell lines generated from a distinct tumor. This gene dependency database could lead to the discovery of previously discovered altered pathways that predict medication response in tumors.

When compared to other publicly available pathway-level methods that simply examine gene sets, the directionality of signaling pathways may improve the accuracy and robustness of the study. PathOlogist, a freely available tool that analyzes not only the individual protein–protein interactions in each pathway, but also the type of interaction and the directionality of signaling pathways, may improve the accuracy and robustness of the study [64].

Before such biomarkers become clinically relevant, they must be reproducible across multiple platforms and datasets. According to previous research, pathway-based biomarkers, rather than individual gene-based biomarkers, appear to have a higher reproducibility [65]. This is because biological processes frequently affect multiple genes simultaneously, allowing the extraction of a more constant metric from the expression pattern of individual genes.

As a quantitative, high-throughput technology, metabolomics has various advantages for developing predictive, diagnostic, and prognostic breast cancer markers. Since the Warburg discovery, already summarized above, the role of glycolytic flux in the oncogenesis of breast cancer has been demonstrated in various studies. Given cancer’s metabolic reprogramming, it is fair to assume that some of these changes will be stable and adaptable to quantitative assessments for diagnostic and prognostic purposes [66]. A metabolomic technique has been utilized in several studies to discover breast cancer changes that could be used for disease stratification, accurately identifying the types of tumors responding to certain therapies. Metabolic profiling is used to identify possible biomarkers of breast cancer or the metabolic fate of administered drugs [67]. Metabolomic analysis can assess metabolic profiles between cancer patients and healthy people or, assess the differences before and after initiation of the disease or progression of the disease. By comparing wild-type cancer controls, the impacts of mutations or knockouts that potentially minimize or eliminate the possibility of metastasis can be detected.

If metabolomics successfully finds breast cancer biomarkers for certain subtypes or medication responsiveness, it will give non-invasive approaches to precisely describe the characteristics of a patient’s cancer in the clinic [66]. Identifying oncometabolites will also help in the targeting of metabolic pathways that promote cell survival and treatment resistance. Efficient data management and analysis tools for large data sets, as well as a better method of incorporating metabolomics data with transcriptomics and proteomics information to translate high-throughput data to clinical diagnosis, can all help to speed up the translation of new laboratory findings to the clinic.

### 3.2. Pharmacomicrobiomics, Using the Microbiome to Predict Resistance and Enhance Immunotherapies

Pharmacomicrobiomics is an innovative field investigating the interplay of microbiome variation and drugs pharmacodynamics to enhance therapeutic efficacy and abrogate side effects. Pharmacogenomics has been at the vanguard of research into how a person’s genetic background influences drug response variability and toxicity. The gut microbiome, commonly known as the second genome, has lately been identified as a critical factor in this area. Combining pharmacogenomics and pharmacomicrobiomics will lay the groundwork for significant advances in personalized medicine. BugBase and PICRUSt2 [68,69], for example, are bioinformatics tools for studying microbiome function. The goal is to use broad phenotypic characteristics such as “anaerobe” and “glucose using” to quantify the functional makeup of commensal bacteria. Other programs, such as QIIME 2 [70], seek to provide a framework for microbiome multi-omics or concurrent exploration of which microorganisms are present. Their metabolic activities and functional possibilities go beyond microbiome connections to microbiome processes.

The gut microbiota has an essential role in carcinogenesis and anticancer therapy response; therefore, is only suitable for the microbiome to become one of the several existing hallmarks of cancer. Researchers have gradually discovered that both normal breast tissue and breast cancer tissue contain a diverse array of microbiota, existing a link between the breast microbiota and breast carcinogenesis and between therapeutic response and drug resistance [71,72]. The microbial presence on breast cancer is easy to understand due to the high adipose composition, lymphatic drainage, and extensive vasculature of the breast, which makes it a favorable environment for bacterial location and growth. Proteobacteria, Firmicutes, and Bacteroides positively correlate with fatty acid metabolism by-products and fatty acid biosynthesis in mammary tissues [73].

**Figure 1:**
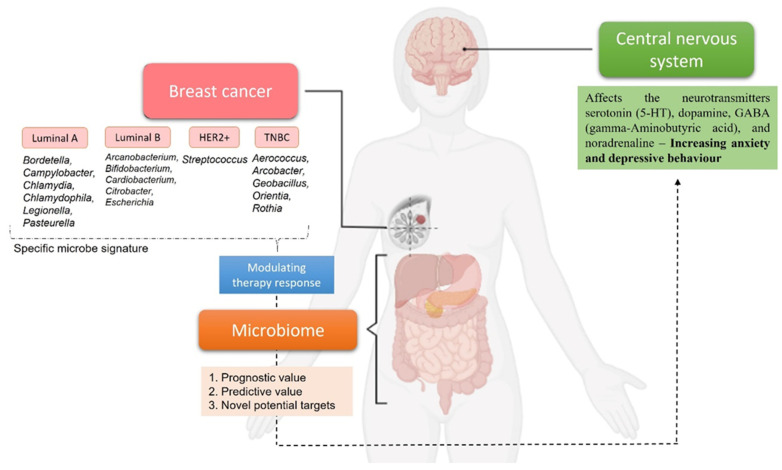
Example of distinct microbial signatures demonstrated by Banerjee et al. that can be associated with different breast cancer subtypes [72]. Once a definite breast cancer microbiome profile is defined, each signature will have a prognostic/predictive value or can be use as potential target to modulate therapy response. The relationship between the microbiome with breast cancer and the central nervous system highlights the importance of these systems for optimal cancer treatment. Created with BioRender.com, last accessed in 10 February 2022.

The interesting part of the microbial presence in breast cancer is the possibility to distinguish microbiome signatures when comparing either normal breast tissue with breast cancer tissue or the differences in tissues representing different breast cancer subtypes [74]. Sphingomonas were found in high concentrations in normal breast tissue, while Methylobacterium were found in high concentrations in breast cancer tissue. Sphingomonas are abundant in normal breast tissue and may impact breast cancer progression in various ways, including estrogen metabolism and activation of TLR that reduce breast cancer development [75]. About two-thirds of estrogen receptor ER+ breast cancer tissue is colonized by Methylobacterium [76]. When comparing the community microbiome of breast cancer patients and healthy participants, microbial diversity is crucial, and a microbiome signature can be associated to each breast cancer subtype. In a study by Banerjee et al., it was possible PathoChip to use to determine a microbial signature for each breast cancer molecular subtype, which is represented in Figure 1. Bordetella, Campylobacter, Chlamydia, Chlamydophila, Legionella, and Pasteurella were abundant in luminal A tumor tissue. In contrast, Arcanobacterium, Bifidobacterium, Cardiobacterium, Citrobacterium, and Escherichia coli were found in abundance in Luminal B tumor tissues. Most hormone-positive breast cancer tissues reported to have a decreased presence of the genus Methylobacterium in comparison to healthy breast tissue. In HER2 tumors, Streptococcus was most abundant, and TNBC tissue was shown to harbor Aerococcus, Arcobacter, Geobacillus, Orientia, and Rothia at the highest level compared to healthy breast tissue. In addition, ER+/PR+ and HER2+ tumors share a similar microbial signature [72]. In the article by Dieleman et al., is possible to find an overview of studies analyzing breast cancer microbiota composition and all these studies reveal that the microbiota makeup of healthy breast, normal adjacent, and tumorous breast microbiota differ significantly. It appears that a breast microbiome exists and that it may change over time as breast cancer progresses. A definite breast cancer microbiome profile, on the other hand, has yet to be developed. Further microbial signatures’ qualitative and quantitative analysis may provide useful diagnostic and prognostic information for breast cancer patients, as well as clues for the development of novel treatment regimens [77].

Furthermore, cancer patients have a lower total microbiome diversity, with research showing that breast cancer patients have a lack of diversity and an abundance of pathogens, with relative increases in Enterobacteriaceae, Bacillus, and Staphylococcus species [78]. It is worth mentioning that some of these species have members who can cause double-stranded DNA breaks in breast cancer cells. There is a considerable difference in the microbial communities of malignant and benign breast tissue samples, with cancer being related with a rise in particular bacteria that are typically less prevalent. Overall, the microbiome of women with breast cancer contrasts from that of healthy women in terms of the number, kinds, abundance, and quality of species (e.g., at the metabolic and immunological levels). As a result, the microbiome has the potential to be an additional risk factor and forecaster of breast cancer, impacting therapy options [79].

#### 3.2.1. Factors Influencing Gut Microbiota Composition

During anticancer treatment, the community structure of gut microbiota is also affected by diet, surgical interventions, antibiotics, prebiotics, probiotics, stress, hygiene, among others. Many of these factors contribute to mechanisms of cancer regulation; (1) dysbiosis, a perturbation of the microbial community, which disturbs the symbiotic association with the host, leading to cancer initiation and progression by the attachment and invasion of pathogenic bacteria to the epithelium; (2) anti-tumor effect via effector cell recruitment and the activation of tumor-specific cytotoxic cells; (3) immunosuppressive action, dampening the antitumor immunity; and (4) microbial modifications of dietary substrates producing potentially carcinogenic products that can contribute to genotoxicity and inflammation. For example, dysbiosis, which is common in non-responders to PD-1 therapy, can cause inflammation and halt T cell differentiation into CD8+ effector cells and has been linked to a significant decrease in Sphingomonas proportion [80,81]. Oral Bifidobacterium can improve PD-L1 efficacy by increasing tumor cell control and contributing to interferon production by CD8+ tumor-specific T cells. It also increases the activation of intratumoral dendritic cells [82].

Diet, antibiotics, prebiotics, and probiotics are the most studied contributors. Diet has been identified as a modifiable breast cancer risk factor. Identifying at-risk people by highlighting predicted diet-related indicators would be extremely beneficial to public health. The ingestion of animal fats, dietary fiber, and vegetables is linked to different patterns of gut microbiota composition. For example, gut microbiota composition and gut bacterial beta-glucuronidase activity are influenced by a high-fiber diet. This inhibits estrogen deconjugation and reabsorption while boosting estrogen fecal excretion, resulting in lower estrogen levels. The synthesis of bioactive metabolites by estrogen-dependent and non-estrogen-dependent processes of the gut microbiota influences breast cancer development [83].

Previous studies show that antibiotic exposure reduces the diversity and richness of some bacterial communities and changes the balance of the gut microbiome, which has been linked to an increased risk of breast cancer. Antibiotics have the potential to harm both the target pathogen and the human host’s commensal residents. The influence on non-target microbial communities is determined by the antibiotic employed, its method of action, and the community’s level of resistance [84].

Various in vitro and laboratory animal-based studies show that prebiotics and probiotics can be used in the prevention and management of several cancers. Probiotics act as functional food and can be coupled with different drugs to increase/modify the efficacy of treatment or reduce its negative effects. The ability of probiotics to restore mucosal barriers, change the composition of microorganisms in the body positively and influence the immune response promotes digestion and reduces stored fat, as well as levels of unnecessary and toxic substances, which is extremely valuable. *Lactobacillus*, *Lactococcus*, *Bifidobacterium* and *Enterococcus* are common bacterial probiotics. The consumption of probiotics containing live bacteria, such as *Lactobacillus* spp., alters the composition of the gut microbiome and has been demonstrated to reduce fecal beta-glucuronidase activity [85]. This is due to a drop in estrogen levels, which lowers the risk of breast cancer. Nonetheless, the use of probiotics must be cautious since clinical research have found that some bacteria utilized as “probiotics” in fermented food products are not effective. Some probiotic strains may be responsible for some side effects including systemic infections, deleterious metabolic activities, gene transfer, and excessive immune stimulation in immunocompromised subjects [86].

The beneficial effects of probiotics on human health can be intensified by the presence of prebiotics, nondigestible substances that are degraded by gut microbiota. Prebiotics protect against cancer by modulating colonic pH, fecal bulking, xenobiotic metabolizing, carcinogen binding to bacteria, enzyme modulation, gene expression modification in the feces, and immune response regulation [85]. More in vivo and clinical trials are needed to confirm the significant role of the use of pre- and probiotics as well as their metabolic products in cancer prevention and treatment, since most of the positive results provided by pre- and probiotic treatments are limited to experimental settings. To minimize side effects associated with probiotics, short- and long-term effect studies with the aim of methodology standardization are essential.

In addition, the type of drug administration is of importance. Drugs taken orally are absorbed into the bloodstream through the epithelial membrane. The solubility, stability, and permeability of the medicine, as well as its metabolism by body and gut microbial enzymes, impact the efficiency of this procedure. The liver and gut bacteria have different metabolic reactions: the liver creates hydrophilic by-products predominantly through oxidative and conjugative metabolism, whereas the gastric microbiota produces hydrophobic by-products primarily through reductive and hydrolytic metabolism [87]. Because the microbiome influences host immunity, it may have a significant impact on the responsiveness and toxicity of several cancer treatments. Pattern recognition receptors (PRRs) bind to bacterial components known as pathogen-associated molecular patterns and instruct the innate immune system to recognize microorganisms; therefore, specific microbial mechanisms recognized by PRRs in the breast can induce a tumor-inhibiting inflammatory response, contributing to the recruitment of tumor-killing cells [88]. PRRs that are expressed by macrophages, DCs, and NK cells include toll-like receptors (TLRs), nucleotide-binding oligomerization domain (NOD)-like receptors, and C-type lectin receptors. When TLRs bind to microbial structures including lipopolysaccharide, peptidoglycan, flagella, or microbial DNA or RNA, they produce inflammation. Depending on the TLR subset, cancer type, and immune cells participating in the tumor, TLR activation can either promote or prevent tumor growth [89]. TLR5 was substantially expressed in mouse xenograft breast carcinomas. The TLR5 ligand Salmonella typhimurium flagellin [90] induced the release of proinflammatory cytokines and chemokines, which had anticancer action.

Resistance to treatment, whether intrinsic or acquired, continues to be a major hurdle to effective breast cancer treatment. Changes in the expression or mutation of a drug target, tumor heterogeneity, decreased blood supply to the tumor, and the TME’s ability to inhibit immune evasion are all factors that contribute to drug resistance [91]. Biomarkers that can predict the therapeutic sensitivity of breast tumor cells are badly needed so that therapy and dose can be altered as needed. We can develop markers to determine therapeutic efficacy by correlating the composition of the breast microbiome with the availability and cytotoxicity of anti-cancer medicines. Identifying which microbial composition promotes a good antitumor response improves therapeutic efficacy.

Microbial metabolism may induce significant side effects, requiring the suspension of chemotherapy. Irinotecan-induced mucositis, for example, causes severe, dose-limiting diarrhea in up to 30% of patients [92]. As a result, microbiome profiling may help identify patients at risk of irinotecan-induced mucositis, while microbiota manipulation could lead to new therapeutic alternatives. Immunotherapeutic strategies aim to reduce immunosuppression in cancer patients by blocking coinhibitory molecules in the TME. Immune checkpoint inhibitors, such as anti-PD-1 and CTLA-4 antibodies, work by preventing T-cell inhibition to improve immunotherapeutic efficacy. It is necessary to acknowledge that the TME is a promising target for improving immunotherapy responsiveness, which modulates protumor inflammation. The impact of gut microbiota on immunotherapeutic effectiveness has previously been described. However, it is unclear whether intratumoral breast microbiota influences immunotherapeutic efficacy or not, because activating TLRs by bacterial products promotes immune cell maturation and priming.

Some bacteria, such as Faecalibacterium prausnitzii, Roseburia intestinalis, and Anaerostipes butyraticus, use fermentation to break down complex carbohydrates and produce short-chain fatty acids (SCFAs: acetate, propionate, butyrate), which modulate host immune cells and provide a carbon source [93]. SCFAs modulate numerous cancer hallmarks, being important regulators of immune cell activation, recruitment, and differentiation, such as neutrophils, macrophages, DCs, and T-lymphocytes. Through the activation of macrophages and DCs, SCFAs can have anti-inflammatory effects on host immune cells, regulating the expression of pro-inflammatory cytokines such as tumor necrosis factor (TNF-), interleukin-6 (IL-6), and interleukin-12 (IL-12) [94]. Butyrate has been demonstrated to stimulate: the Treg cell differentiation in vitro and in vivo; the anti-inflammatory forkhead box protein P3 (Foxp3), which is necessary for the suppression of inflammatory reactions [95]; and T-helper (Th) cell cytokine profiles, supporting intestinal epithelial barrier integrity, which might help limit mucosal immune system exposure to luminal microorganisms and prevent aberrant inflammatory responses. *Bifidobacteria* species produce acetate, a SCFA that modulates intestinal inflammation. SCFAs reduce inflammation in the stomach through a variety of methods, including HDAC inhibitors, histone acetyltransferase activity stimulation, and HIF stabilization [96,97]. 

Aside from direct effects of organ-specific microbiota on local tissue, the gut microbiota can influence breast cancer development by indirect pathways such as enterohepatic estrogen recycling, bile acids, and microbial interaction with the innate and adaptive immune systems. Estrogens are conjugated in the liver and transported to the gut by bile excretion. Bacteria with beta-glucuronidase enzymatic activity [98] are found in the gut and are capable of deconjugating conjugated estrogen, which is reabsorbed into the circulation, resulting in greater estrogen exposure throughout the body and an increased risk of breast cancer. The deconjugation of conjugated estrogens is carried out by bacterial β-glucuronidases, which are found in the subsequent bacterial genuse: Collinsella, Edwardsiella, Alistipes, Bacteroides, Bifidobacterium, Citrobacter, Clostridium, Dermabacter, Escherichia, Faecalibacterium, Lactobacillus, Marvinbryantia, Propionibacterium, Roseburia, and Tannerella [99]. Estrogen reactivation allows them to be reabsorbed and increase serum estrogen levels, and estrogen-induced changes in mitochondrial gene expression have been linked to estrogen-induced carcinogenesis [100]. β-glucuronidase activity and gut-derived metabolites can be modulated by diet. Poor dietary choices and obesity benefit the growth of certain bacteria species such as *Fusobacterium nucleatum*. This bacterium can elevate the host’s lymphocytes by killing mature lymphocytes. A low lymphocyte count in cancer patients is related to poor prognosis.

Moreover, the main receptors of SCFAs are the free fatty acid receptors (FFARs) that are not only located on the cancer cells, but also on stromal cells (e.g., adipocytes) [101]. SCFAs can have positive [102] and negative [103] effects in breast cancer. As a result, the manipulation of SCFA levels in the intestinal tract caused by changes in microbiota structure could be considered for cancer treatment or prevention. Lithocholic acid is a secondary bile acid that is synthesized from chenodeoxycholic acid (CDCA) and ursodeoxycholic acid (UDCA) by bacterial 7-dehydroxylation [104]. The human body’s capacity and the microbiome to synthesize LCA are largely reduced in breast cancer, which is the most dominant in early stages (stages 0 and 1). In breast cancer, serum lithocholic acid levels were found to be adversely linked with the Ki67 labeling index [105]. Bile acid transformation is carried out by anaerobic bacteria, primarily Clostridiales [106]. The bile acids found in the breast come from the gut, the bacterial enzymes LdcC and CadA produce cadaverine from lysine [107]. Although human cells can synthesize cadaverine, bacterial cadaverine production outnumbers human biosynthesis. Cadaverine causes cells to become more glycolytic since it has been demonstrated that they reduce cellular oxygen consumption, which indicates OXPHOS activity [108].

Detailed microbiome signatures and diversity may be a useful biomarker for diagnosis and prognosis in patients with breast cancer. The gut microbiota can influence the adverse effects and efficacy of anticancer drugs in individuals with breast cancer through immune modulation and anticancer drug metabolism.

#### 3.2.2. The Microbiome Intervein with Neurophysiological Function

The microbial habitat of the digestive tract is the most heavily inhabited in the body. In addition to fungi, viruses, and archaea, the bacteria that make up much of this ecosystem are vital for immunological, metabolic, psychological, and cognitive function. Dysbiosis can lead to abnormal neurophysiological function and behavior, such as anxiety and depression [109]. The neurotransmitters serotonin (5-HT), dopamine, GABA (gamma-Aminobutyric acid), and noradrenaline are affected by the gut microbiota [110]. For example, SCFAs have immunomodulatory properties and can interact with nerve cells by stimulating the sympathetic and autonomic nervous systems via G-protein-coupled (GPR) receptors 41 (GPR41) and GPR43. SCFAs also cross the BBB and affect gut–brain hormonal communication by regulating the release of gut peptides from enteroendocrine cells (EC). SCFAs have recently been shown to regulate EC cell production of gut-derived 5-HT [111]. This is an important topic since neurotransmitters, notably serotonin, which regulates mood, are implicated in the neurological dysregulation seen in depressed patients and cancer survivors who typically develop comorbid depression after a cancer encounter. Therefore, by ensuring that breast cancer patients dispose of healthy gut microbiota, we create an opportunity to improve the selected therapeutic and after treatment lives of survivors.

Because microbes in the human gut translate to a wide range of enzymes, the gut microbiome is becoming a key player in personalized medicine. This greatly expands the catalogue and function of metabolic reactions in the human body that can be involved in xenobiotic metabolism, including dietary components and drugs [112]. As a result, pharmacomicrobiomics can be considered for clinical applications such as using a combination of microbiome and genetic profiles to better predict a person’s medication reaction and/or modifying the gut microbiome to improve drug efficacy on a per-person premise. To further understand the underlying causes and mechanisms, a systems-based approach and specialized drug testing procedures are necessary. Due to the complexity and inter-individual variability of human microbiota, identifying microbial colonization profiles—especially associated with various illnesses and the characterization of microbial metabolic pathways connected to health and disease states—remains a problem. The human microbiota interacts with the immune system at multiple levels. Changes in this crosstalk may involve the host’s pathophysiological mechanisms, which can then be used to develop clinical therapies for some immunological disorders. This could lead to the development of potential biomarkers, which would allow for the implementation of personalized healthcare strategies and the identification of new tools for prevention, screening, and treatment.

## 4. Conclusions

In breast cancer treatment, the goal of precision medicine continues to hold promise for more specialized and tailored care. Every day, the treatment of breast cancer advances towards more personalized care, from the discovery of endocrine and HER2-focused medicines to multigene arrays in chemotherapy for more specific patient selection, to radiomics and genetic subtyping. The goal for treating cancer patients is to develop real-time biomarkers for cancer that will allow real-time management of the disease, similar to diabetes, where the individual can tell what their specific needs are by analyzing blood sugar levels. Because cancer is so complex, “one-size-fits-all” treatments may not be practical, but it may be possible to quantify the benefits of treatment before months have gone by.

Today’s personalized cancer therapy incorporates data from a wide range of diagnostic tests and patient history to determine the best treatment for each patient, but lacks real-time measurements of effectiveness and resistance to reduce relapses. Because each patient’s response to treatment is heavily influenced by their genomic background and/or the genetic makeup of the tumor, the majority of current approaches for cancer patient stratification rely on genetic tests. However, the genetic makeup of a tumor does not show us its full identity. If we consider the TME, the immune system, and the microbiome, we can have a better foundation for guiding diagnosis, therapy selection and monitoring, and patient follow-up. This comprehensive approach will represent a major step forward by analyzing the metabolism of immunotherapy drugs in different subtypes and conjugating that information with the immunometabolism and microbiome response (without neglecting the information provided by genetics) in order to accurately identify breast cancer patients who are either excellent candidates or unsuitable for certain medications.

## Figures and Tables

**Table 1:** In different breast cancer subtypes there is a link between their receptor profile, subtype prevalence, subcategories, and the major infiltrating immune cell pattern. Immune cells are distributed differently in each subtype. The words in blue denote specific immune cells that are associated with a good prognosis, the words in red denote the infiltrating signature that is associated with a poor prognosis, and the words in green denote a lower proportion of immune cells that is also associated with a good prognosis.

Breast Cancer Subtype	Receptor Profile	Subtype Prevalence	Subcategories	Prognosis	Immune Cell Patterns
Hormone positive	ER+ or PR+	70%	Luminal A	When compared to other subtypes, it grows more slowly and is less aggressive.	Nk, Neutrophils Tregs, TAMs 1 and 2, Mast cells TCD8+, TCD4+, B lymphocytes
			Luminal B	Because it has a higher grade than luminal A, it is linked to a worse prognosis.	
HER2 positive	HER2+	20%	-	Poor prognosis and aggressive disease progression	Tregs, Neutrophils, DCs, Mast cells, Tγδ
Triple-negative breast cancer	Er−, Pr−, and HER2−	10%	Basal-like 1 and 2 (BL-1, BL-2), immunomodulatory (IM), mesenchymal (M), mesenchymal stem cell-like (MSL), and luminal androgen receptor (LAR)	It has the worst prognosis. TNBC is extremely common among black women and those who have a BRCA1 gene mutation.	Tregs, TAMs 1 and 2, Mast cells TCD8+, TCD4+, DCs

**Table 2:** A summary of the metabolic changes linked to drug resistance in cancer.

Pathways Associated with Metabolism	Target Proteins/Enzymes or Metabolites	Therapy
Glycolysis	GLUT1, Hexokinase, LDHA, Pyruvate kinase, SGLT-2	Lapatinib, Paclitaxel, Trastuzumab, 2-deoxy-D-glucose, Dapagliflozin, Oxamate and Tamoxifen
Fatty acid synthesis	FASN	Adriamycin, Omeprazole, Conjugated linolic acid, Orlistat, Fasnall, Cerulenin and C75
Redox metabolism	GCLC	Tamoxifen
Mitochondrial energy metabolism	ERRα, NQO1	Lapatinib, Tamoxifen
TCA cycle	Pyruvate dehydrogenase kinase (PDK3)	siRNA, Metformin

**Table 3:** Biomarkers classified into categories, and examples of each category for breast cancer.

Predictive Biomarkers	Predict Response to a Therapy	A Breast Cancer Patient with Extra Copies of the HER2 Gene Will Respond Favorably to the HER2 Inhibitor Trastuzumab
Prognostic biomarkers	Predict patient outcome	Ki-67 and proliferating cell nuclear antigen overexpression; estrogen receptor (ER) and progesterone receptor (PR) overexpression; transforming growth factor- (TGF-); apoptotic imbalance indicators, including bcl-2 overexpression and an elevated bax/bcl-2 ratio; changes in differentiation signals, such as c-myc and related protein overexpression; loss of differentiation markers, such as TGF-II receptor and retinoic acid receptor; and changes in angiogenesis proteins, such as VEGF overexpression, are all instances.
Diagnostic biomarkers	It helps clinicians to identify a subtype of cancer accurately	Carbohydrate antigen 15-3 (CA15-3); circulating DNA (ctDNA) and RNA (e.g., micro RNAs); circulating tumor cells and exosomes
Risk assessment biomarkers	Predicts the patient’s risk of developing a malignancy	Pathogenic mutations in BRCA1 and BRCA2 is a risk factor for developing breast and ovarian cancer
Cancer recurrence monitoring biomarkers	Surveillance marker to monitor recurrence of cancer	Chemokine receptor 9 (CCR9); miRNAs by downregulating E-cadherin and thus affecting EMT and breast cancer cell metastasis; non-cancer cell components
Biomarkers Involved in Cancer Drug Resistance	Identifies possible markers for drug resistance	Estrogen Receptor Alpha (ESR1) Mutation; miRNA; circRNA

## Data Availability

Not applicable.
